# Pilbara Craton Soil as A Possible Lunar Soil Simulant for Civil Engineering Applications

**DOI:** 10.3390/ma12233871

**Published:** 2019-11-23

**Authors:** Janusz Kobaka, Jacek Katzer, Paweł K. Zarzycki

**Affiliations:** 1 Faculty of Civil Engineering, Environmental and Geodetic Sciences, Koszalin University of Technology, 75-453 Koszalin, Poland; 2 Faculty of Geodesy, Geospatial and Civil Engineering, University of Warmia and Mazury in Olsztyn, 10-720 Olsztyn, Poland; 3 Faculty of Civil Engineering, Environmental and Geodetic Sciences, Koszalin University of Technology, 75-453 Koszalin, Poland; pkzarz@wp.pl

**Keywords:** lunar soil, simulant, LSS, Pilbara Craton, concrete

## Abstract

Recent fast development in lunar exploration exposed a lack of lunar soil simulant (LSS) fit for civil engineering applications. Permanent human presence on the Moon will be associated with significant construction efforts. Adequate technologies and building materials have to be developed and tested prior to setting the actual building site on the Moon. Current LSSs were created for non-civil engineering purposes, thus they are very expensive and available in limited amounts. In the paper, the authors proved that Pilbara Craton soil is a suitable material for the creation of an affordable LSS for civil engineering applications. The main tool of the conducted study was principal component analysis (PCA).

## 1. Introduction

**Figure 1:**
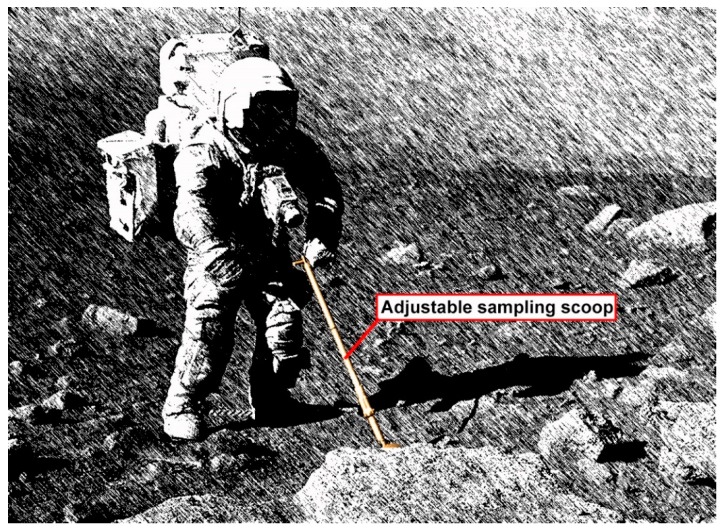
Apollo 17 mission—geologist-astronaut Harrison Schmitt retrieving lunar soil samples (picture by J. Kobaka, created based on original NASA photographs, sourced from Wikimedia Commons).

After 50 years, since the first human landing on the Moon, the scientific world still struggles to create high-quality lunar soil simulants (LSSs). Apollo 11 brought back to Earth only 22 kg of lunar soil, including 50 rocks. The amount of lunar soil brought back by subsequent Apollo missions was increasing constantly, but still the fifth and last lunar mission (Apollo 17) brought back only 111 kg of lunar soil. The relatively small amount of collected samples was caused by multiple limitations, such as the short time of the actual presence on the Moon and conducting other scientific experiments. Nevertheless, the laborious character of the very process of lunar soil collection, which is presented in Figure 1, was a key obstacle. Altogether, 382 kg of lunar rocks, core samples, pebbles, sand and dust were acquired by the Apollo programme. Due to a very limited amount of lunar soil brought back from the Moon and directly available for research programmes, a need for the creation of LSSs emerged very quickly. Worldwide, numerous research teams tried to compose material in order to closely model lunar soil. This international research effort resulted in the creation of numerous LSSs available commercially and quasi-commercially [1]. The majority of proposed LSSs have proved to be challenging to manufacture, especially on a quasi-industrial scale [2,3,4,5,6].

Other LSSs have not stood up to research teams’ expectations and over time proved inadequate for numerous testing tasks [7]. This issue is partially associated with the limitations of the harnessed methodology of testing the geotechnical characteristics of lunar soils [8]. One should also remember that almost all created and scientifically described LSSs were engineered for a very specific research programme. In any given case, the precise mirroring of particular properties (or characteristics) and omitting of others of lunar soils was a conscious choice of a research team [2,3,5,6].

Some research programmes require tiny volumes of LSSs, which are ultimately changed or destroyed during tests [9,10]. On the other hand, during the development of lunar autonomous vehicles and human-driven rovers, there is a need for significant amounts of LSSs. Testing lunar vehicles does not influence or damage utilized LSSs in any way, enabling their reuse for other purposes [1]. In comparison, civil engineering research programmes are very demanding as to LSSs. They require large volumes of LSSs, which are ultimately destroyed during tests. The lack of affordable LSS available in large volumes is a key obstacle in creating lunar cement-like binders, concrete-like composites and full-scale construction technologies [11]. The only LSSs matching the criteria of affordability and availability in large volumes were GRC-1 and GRC-3 [12,13,14]. In the authors’ opinion, there is an urgent and growing need for new LSSs for solely civil engineering research programmes. Such LSSs with key granulometric [15,16] and chemical characteristics crucial for the development of the future civil engineering industry should be created. The aim of this study is to find a suitable and easily available material for such an application, because future Moon or Mars habitats are envisioned to be built from lunar and Martian in situ resources [17].

## 2. Key Properties of Needed LSS

**Figure 2:**
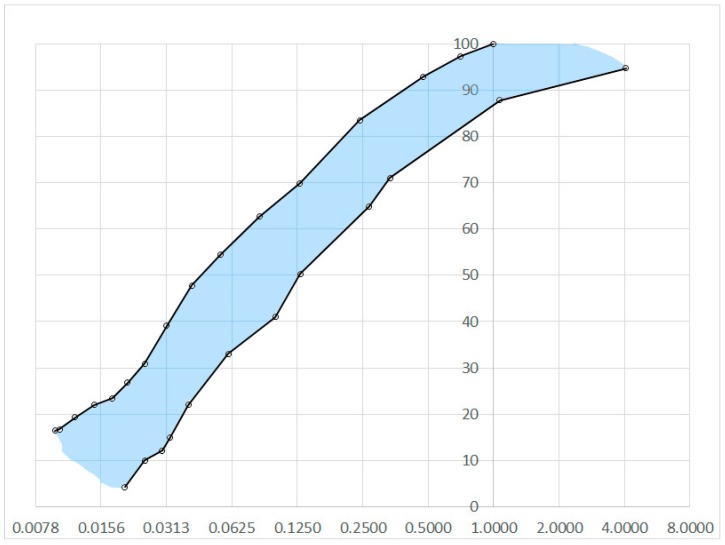
Grain size distribution range of Apollo 14 mission samples. Based on data sourced from [18].

**Figure 3:**
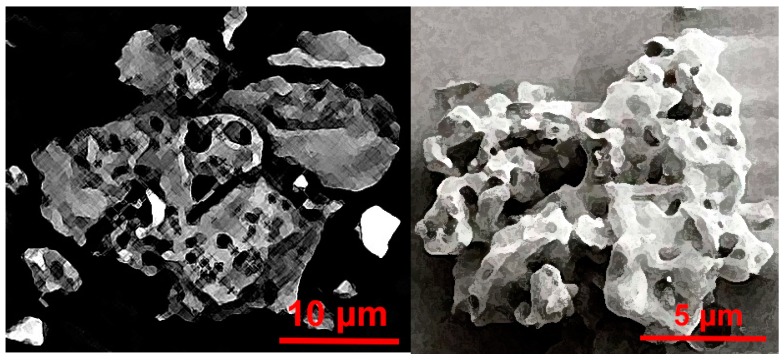
Shapes of lunar soil grains (pictures by J. Kobaka, created based on original NASA photographs, sourced from Wikimedia Commons).

**Figure 4:**
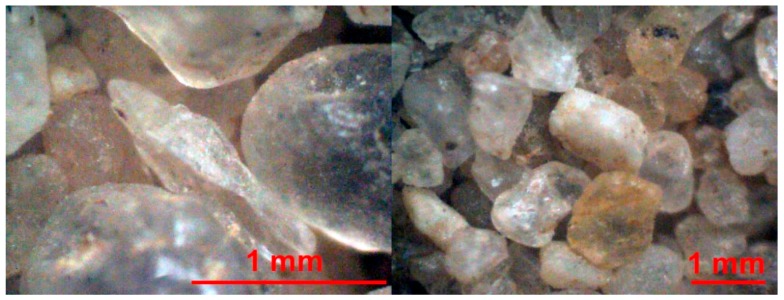
Grains of post-glacial sand in microscopic magnification (photos by J. Katzer and J. Kobaka).

While creating LSSs, it is very important to keep in mind multiple specific differences between ordinary lunar and Earth soil particles. After thorough study, the authors differentiated three main characteristics that define LSSs suitable for civil engineering application. The first and, at the same time, the easiest to achieve is its granulometric composition. The particle size distribution of the lunar soil indicates a high volume of very fine grains. The LSS grain size distribution should be contained within the range of the natural lunar soil presented in Figure 2. The granulometric composition of a LSS closely mirroring natural lunar soil is quite easy to achieve. Grinding processes are very common in numerous industries. The creation of material characterized by a certain grain size distribution, using grinding and sieving techniques, does not create serious technological problems. The second key characteristic is the shape and roughness of the surfaces of lunar soil particles. Due to the lack of an atmosphere on the Moon and the erosion processes associated with it, lunar soil particles have sharp edges and an extended shape (see Figure 3), in contrast to Earth soil particles, which are characterized by round edges, which are created by wind, water and glacier erosion activities (see Figure 4). The shapes of lunar soil grains are reasonably easy to mimic (however, not all complex shapes are possible to be produced) with the accuracy needed for the civil engineering applications.

The shape, size and quality of a surface are the key parameters of the aggregate-influencing properties of produced concrete [19]. On the other hand, the creation of aggregates by crushing rocks is quite a common process in civil engineering. In this way, grains characterized by sharp edges and rough surfaces are created. Nevertheless, the commonly achieved geometry of grains is not so sophisticated and expanded as lunar ones. This property is more difficult to achieve than the granulometric composition.

The third key characteristic of the needed LSS is its chemical composition. This characteristic is equally important as the former two but, at the same time, it is the most difficult to achieve. The chemical composition of aggregates used for ordinary concrete production directly influences its strength, creep, shrinkage, durability and vulnerability to corrosion. One can expect similar relations in the case of lunar concrete-like composites. Thus, the precise mirroring of lunar soil’s complex chemical composition is essential for LSSs for civil engineering applications. The varied quantities of the numerous minerals contained in lunar soil cause difficulties in the effective comparison and assessment of LSSs and lunar samples. The method of LSS quality assessment proposed by Zarzycki and Katzer [20,21], based on principal component analysis (PCA), is currently the most reliable one. The authors decided to use this method to analyze existing and newly proposed LSSs.

## 3. PCA Quality Determination of Available LSSs

PCA has been gaining popularity as it shows strong patterns from complex datasets. This is a powerful analytical tool that finds internal correlations within a set of data and develops a statistical representation of these datasets [22]. PCA produces an optimal presentation of multivariate data by projecting raw data into the space defined by the eigenvectors of the data variance–covariance matrix (Johnson, Smith and Adams, 1985).

The main purpose of PCA [23] is to:

- Identify hidden patterns in a data set;
- Reduce the dimensionality of the data by removing the noise and redundancy in the data;
- Identify correlated variables.

PCA reduces the dimensionality of multivariate data into two or three principal components, which can be visualized graphically, with minimal loss of information [23]. Kaiser [24] recommends taking into account principal components characterized by eigenvalues (values measuring the amount of variation retained by each principal component) such as λ*i* > 1, which usually reduces the number of main components to two or three. Considering two main components—factor 1 (F1) and factor 2 (F2)—the results can be presented as a two-dimensional plot. If F1 is the first main component, F2 the second main component and *X* the matrix of observed variables, then F1 and F2 are the first two columns of the matrix F as the result of two matrices multiplication:

*F* = *Z*·*B*, (1)

where *Z* is matrix *X* converted into a standard score form (the mean value of the variables is equal to 0 and their variation is equal to 1) and *B* is a factor score coefficients matrix based on correlations.

**Figure 5:**
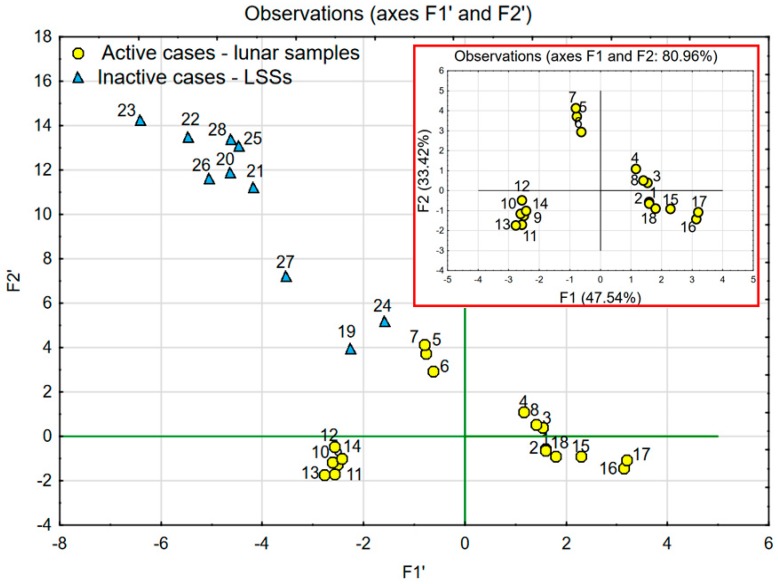
Principal component analysis (PCA) with object grouping in two-dimensional space: Main chart (factor scores F1′ and F2′) presenting analysis of lunar samples in comparison with LSSs as *inactive* cases. Chart in the red rectangle (factor scores F1 and F2)-analysis of lunar samples only.

**Figure 6:**
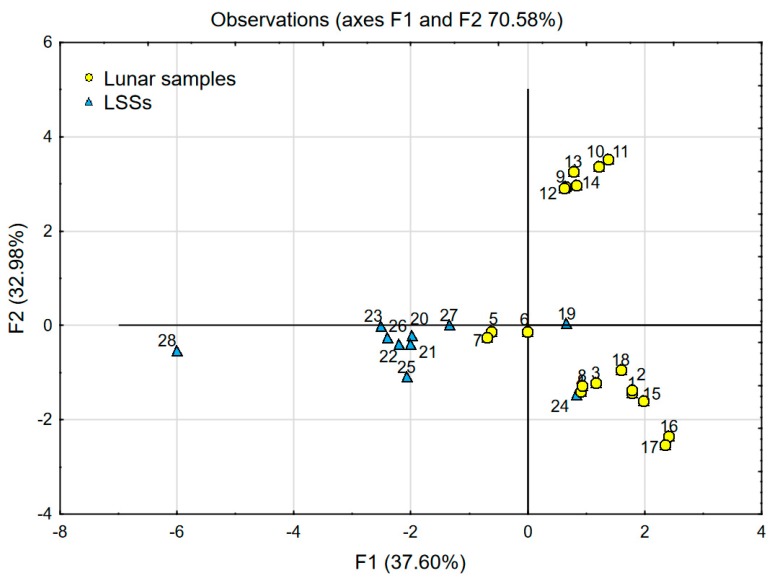
PCA with object grouping in two-dimensional space (factor scores F1 and F2) presenting analysis of lunar samples in comparison with LSSs as *active* cases.

The comparison of lunar soil samples with LSS samples depending on chemical composition was carried out using two scenarios of PCA analysis. The data presented in Table 1 and Table 2 was used for both analyses. The results are presented in Figure 5 and Figure 6.

In the first scenario, PCA analysis was performed using the data of lunar samples (named *active*) described by factors F1 (47,54%) and F2 (33.42%), which together explain over 80% of the variability. The achieved coordinate system was enriched by the data of LSSs (named *inactive*)—see Figure 5. *Inactive* cases (LSS data) did not affect statistical calculations, which were based on lunar soil chemical data, and they play the role of a visible reference.

In the second scenario, PCA analysis was performed using the data of lunar soil samples and LSSs together (see Figure 6). In this case, the two factors F1 (37.6%) and F2 (32.9%) explain over 70% of the variability. The charts presented in Figure 5 and Figure 6 prove that the chemical characteristics of the most popular LSSs described in the literature struggle to correspond with the chemical characteristics of lunar samples. This issue was thoroughly described and discussed in a previous publication [21].

## 4. Pilbara Craton

**Figure 7:**
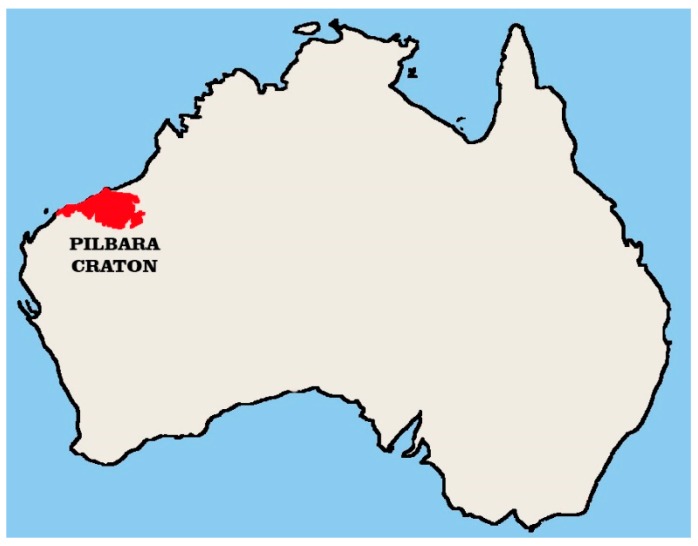
Pilbara Craton location in West Australia (picture by J. Kobaka).

Cratons are the old parts of Earth continental plates. One of them is the Pilbara Craton, located in West Australia (see Figure 7). It consists of three main lithotectonic elements: the East Pilbara Terrane, the West Pilbara Superterrane and the De Grey Superbasin [25]. The age of the Pilbara Craton is assessed to be from 3.53 to 2.93 Ga (giga annum).

**Figure 8:**
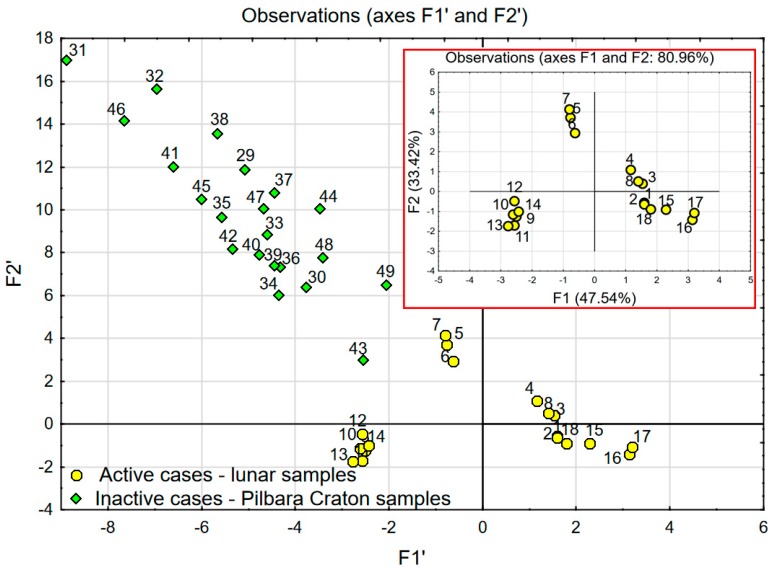
Main chart (factor scores F1′ and F2′) presenting analysis of lunar samples in comparison with Pilbara Craton soil as *inactive* cases. Chart in the red rectangle (factor scores F1 and F2)-analysis of lunar samples only.

**Figure 9:**
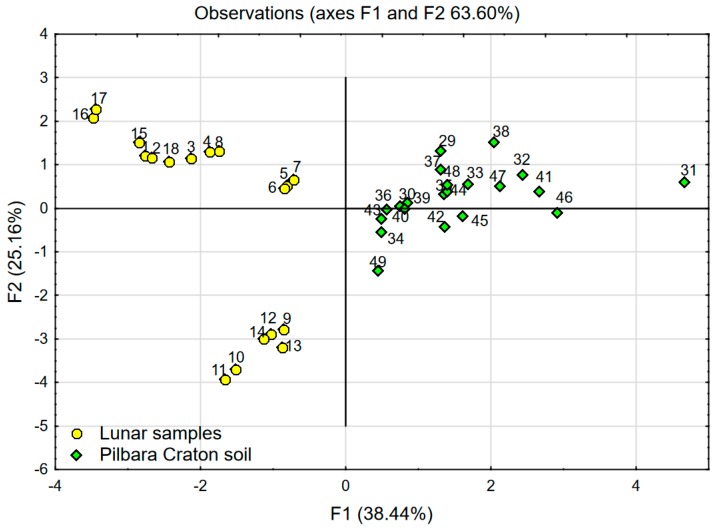
PCA with object grouping in two-dimensional space: Chart (factor scores F1 and F2) presenting analysis of lunar samples in comparison with Pilbara Craton soil as *active* cases.

Some recent studies [25,26] suggest that the cryptic ancient crust might have been formed in the Hadean era (4.5–4.0 Ga). The Moon is thought to have been formed by an impact between Earth and an impactor around 4.5 billion years ago [27]. It is likely that the Pilbara Craton was formed at approximately the same time. Therefore, it is more than justified to compare the mineralogical composition of the Lunar soil with the ancient parts of the Earth’s continental crust. In Table 3, the chemical composition of samples collected in the West Pilbara Craton is presented. This data was used to conduct a comparison with lunar soil samples. The results of this comparison are presented in Figure 8 and Figure 9. As in the case of the comparative analysis of lunar soil and LSSs (see Figure 5 and Figure 6), the comparison was carried out using two scenarios of PCA analysis.

In the first scenario, PCA analysis was performed using data of lunar samples (named *active*). In this case, the two factors F1 (47.54%) and F2 (33.42%) explain over 80% of the variability. The achieved coordinate system was enriched by the data of Pilbara Craton soil (named *inactive*)—see Figure 8. *Inactive* cases (Pilbara Craton data) did not affect statistical calculations, which were based on lunar soil chemical data, and they play the role of a visible reference. In the second scenario, PCA analysis was performed using the data of lunar soil samples and Pilbara Craton soil together (see Figure 9). In this case, the two factors F1 (38.44%) and F2 (25.16%) explain 63.6% of the variability.

## 5. Discussion

One can easily notice significant similarities between the chart presenting the comparison of LSSs with lunar soil samples (see Figure 5 and Figure 6) and the one presenting the comparison of West Pilbara Craton soil with lunar soil samples (see Figure 8 and Figure 9). The position and spacing of LSSs in the chart (points 19–28) are very similar to the case of the West Pilbara Craton (points 29–49). The conducted PCA analysis shows that, in terms of chemical composition, soils of the Pilbara Craton are as good at mimicking lunar soil as any currently available LSS. Keeping in mind that the chemical composition is the most difficult characteristic to achieve in the creation of any LSS, the Pilbara Craton opens brand new opportunities for the development of analogues. The shear amount of available Pilbara Craton raw soils makes it a perfect candidate for the production of LSSs for civil engineering applications. The raw soils are easily accessible and ready for grinding, sieving and other traditional processes used in the aggregate industry. It is feasible to create LSS from Pilbara Craton soil with the chemical and granulometric accuracy needed for the civil engineering applications. Such a LSS would be much cheaper than any current LSSs and available in any needed amount. The shape of the grains should be the main characteristic taken into account during the production of proposed LSS. Full-scale civil engineering research programmes (especially those focused on Lunar concrete) would be finally enabled. One has to keep in mind that before fully committing to harnessing Pilbara Craton soil as a LSS, further testing is required to ensure the similarity between Pilbara Craton soil and lunar soil. Actual lunar soil is exposed to vacuum, radiation and space weathering and these conditions do not resemble that on Earth. Therefore, Pilbara Craton soil (even if it was proven that its origin is the same as lunar soil) can differ significantly in comparison to lunar soil due to numerous giga annums of varied ageing conditions.

## 6. Conclusions

The conducted analysis allows the formation of the following conclusions:

- The chemical composition of Pilbara Craton soil seems to be very similar to current LSSs;
- Pilbara Craton soil should be easily transformed (using traditional civil engineering techniques) into an affordable LSS, which would be available in large quantities;
- LSS based on Pilbara Craton soil would be suitable for a wide range of civil engineering research programmes;
- PCA allows the conduction of complex analyses that give clear results. Such analyses are not feasible using other methods, particularly univariate approaches.

## Figures and Tables

**Table 1:** Lunar sample compositions (wt.%). Based on the data sourced from [20].

Sample No. *	SiO_2_	TiO_2_	Al_2_O_3_	FeO	MgO	MnO	CaO	Na_2_O	K_2_O	P_2_O_5_
1	42.1	7.8	13.7	15.8	7.9	0.2	12	0.5	0.1	0.1
2	42.2	7.8	13.6	15.3	7.8	0.2	11.9	0.47	0.16	0.05
3	46.6	3.6	14.2	15.4	9.7	0.22	10.4	0.43	0.24	0.156
4	46	2.8	12.5	17.2	9.7	0.22	10.9	0.48	0.24	0.156
5	48.2	1.73	17.6	10.41	9.26	0.14	11.25	0.61	0.51	0.53
6	47.3	1.6	17.8	10.5	9.6	0.1	11.4	0.7	0.6	0.156
7	48.1	1.7	17.4	10.4	9.4	0.14	10.7	0.7	0.55	0.51
8	46.95	1.6	12.7	16.29	10.75	0.217	10.49	0.33	0.092	0.16
9	45.35	0.49	28.25	4.55	5.02	0.06	16.21	0.42	0.09	0.1
10	45.2	0.58	26.4	5.29	6.1	0.7	15.32	0.52	0.14	0.12
11	44.65	0.56	27	5.49	5.84	0.7	15.95	0.44	0.13	0.1
12	44.9	0.47	27.7	5.01	5.69	0.242	15.7	0.51	0.22	0.16
13	44.77	0.37	28.99	4.35	4.2	0.07	16.85	0.44	0.06	0.05
14	45	0.54	27.3	5.1	5.7	0.3	15.7	0.46	0.17	0.11
15	41.67	6.52	13.57	15.37	10.22	0.21	11.18	0.34	0.09	0.06
16	39.82	9.52	11.13	17.41	9.51	0.25	10.85	0.32	0.07	0.06
17	40.09	9.32	10.7	17.85	9.92	0.24	10.59	0.36	0.08	0.07
18	42.2	5.09	15.7	12.4	10.3	0.15	11.5	0.24	0.07	0.156

* Lunar samples collected by: 1, 2—Apollo 11; 3, 4—Apollo 12; 5–7—Apollo 14; 8—Apollo 15; 9–14—Apollo 16; 15–18—Apollo 17.

**Table 2:** Lunar soil simulant (LSS) compositions (wt.%). Based on the data sourced from [20].

Sample No. *	SiO_2_	TiO_2_	Al_2_O_3_	FeO	MgO	MnO	CaO	Na_2_O	K_2_O	P_2_O_5_
19	47.5	0.94	15.3	11.12	9.45	0.16	13.3	1.73	0.02	0.02
20	47.5	1.5	15	7.25	9	0.175	10.5	2.75	0.8	0.65
21	49.1	1.48	15.5	9.81	8.48	0.18	10.1	2.46	0.85	0.61
22	46.2	1.85	17.1	11.2	6.87	0.19	9.43	3.33	0.85	0.62
23	47.2	1.81	17.9	10.3	5.93	0.17	10.5	3.53	0.82	0.71
24	42.8	6.77	12.1	16.3	6.19	0.22	11.1	2.22	0.2	0.04
25	49.24	1.91	15.8	11.47	8.74	0.14	7.25	3.08	1.02	0.3
26	49.14	1.91	16.23	13.07	3.84	0.19	9.13	2.75	1.01	0.44
27	52.69	1.01	15.91	12.28	5.41	0.22	9.36	1.9	0.58	0.14
28	69.84	0.78	12.16	8.4	2.03	0.14	2.54	1.07	2.28	0.392

* LSS samples: 19—BIR-1, 20—USGS, 21—JSC-1, 22—JSC-1A, 23—JS-1Af, 24—MLS-1A, 25—CAS-1, 26—FJS-1, 27—MKS-1, 28—Tektite.

**Table 3:** West Pilbara Craton sample compositions (wt.%). Based on the data sourced from [28].

Sample No. *	SiO_2_	TiO_2_	Al_2_O_3_	FeO	MgO	MnO	CaO	Na_2_O	K_2_O	P_2_O_5_
29	50.76	0.79	12.69	3.68	9.91	0.19	8.14	3.19	0.4	0.31
30	50.39	1.80	12.97	4.19	5.73	0.21	10.22	1.95	0.33	0.20
31	65.21	0.61	14.60	1.62	2.40	0.11	4.95	3.62	1.38	0.09
32	51.44	0.91	15.84	2.78	7.35	0.17	7.02	3.70	1.41	0.10
33	54.92	0.65	12.45	3.35	7.12	0.18	9.16	2.25	0.60	0.10
34	48.21	1.01	14.69	3.78	5.6	0.21	12.42	2.11	0.09	0.32
35	51.60	1.35	14.56	3.67	6.81	0.21	9.23	3.12	0.10	0.12
36	48.00	1.22	14.88	4.15	7.45	0.25	10.51	2.53	0.20	0.10
37	50.33	1.06	13.83	3.25	9.13	0.19	8.59	2.53	1.05	0.14
38	54.82	0.91	8.47	3.59	8.97	0.19	8.85	3.10	1.16	0.10
39	49.22	1.38	14.62	4.04	6.82	0.21	10.44	2.49	0.15	0.16
40	49.15	1.32	14.27	3.94	6.74	0.22	10.8	2.63	0.23	0.12
41	56.31	0.79	13.9	3.23	4.45	0.16	7.50	3.15	0.65	0.11
42	51.29	1.62	15.12	3.9	4.31	0.34	8.86	2.70	0.21	0.13
43	49.72	1.26	14.18	4.00	5.72	0.23	10.00	1.33	0.00	0.11
44	49.06	1.26	14.85	3.85	7.12	0.20	8.52	1.52	2.08	0.13
45	51.39	0.40	17.49	1.91	7.26	0.12	10.61	2.89	0.75	0.05
46	54.17	0.90	14.51	1.98	3.19	0.18	8.45	3.4	1.24	0.17
47	55.37	0.49	13.75	2.76	6.71	0.17	8.11	2.05	1.19	0.10
48	51.73	0.27	16.29	2.43	8.72	0.15	7.63	1.80	0.83	0.06
49	39.88	0.89	15.97	3.16	3.91	0.27	12.58	0.07	3.18	0.18

* West Pilbara Craton composition research carried out at Utrecht University and the Free University of Amsterdam, Netherlands. Sourced from [26]—Appendix 5.B.3, p. 235.
